# Benefits of aerobic exercise in myotonic dystrophy type 1

**DOI:** 10.1172/JCI160229

**Published:** 2022-05-16

**Authors:** Samuel J. Mackenzie, Johanna Hamel, Charles A. Thornton

**Affiliations:** Department of Neurology, University of Rochester Medical Center, Rochester, New York, USA.

## Abstract

Myotonic dystrophy type 1 (DM1) is a multisystem trinucleotide repeat expansion disorder characterized by the misregulated alternative splicing of critical mRNAs. Previous work in a transgenic mouse model indicated that aerobic exercise effectively improves splicing regulation and function in skeletal muscle. In this issue of the *JCI*, Mikhail et al. describe the safety and benefits of applying this approach in individuals affected by DM1. A 12-week aerobic exercise program improved aerobic capacity and mobility, but not by the mechanism observed in transgenic mice. Here, we consider the possible reasons for this disparity and review other salient findings of the study in the context of evolving DM1 research.

## Exercise response in DM1

The beneficial effect of exercise in the general population and in many disease states is widely accepted. However, caution must be used in recommending exercise to patients with muscular dystrophies, where muscle fibers may show increased susceptibility to activity-induced injury. For example, muscle injury in Duchenne muscular dystrophy is aggravated even by normal daily activities (1), and the muscle fibers specialized for rapid contraction are particularly vulnerable (2). Defining which type and intensity of exercise is appropriate in muscular dystrophy has been an active area of investigation (3, 4).

In the context of muscle disease where the benefits and harms of exercise need to be carefully weighed, Myotonic dystrophy type 1 (DM1) may be a distinct outlier. The causal mutation is an expanded CTG repeat in the 3′ UTR of the DM1 protein kinase (*DMPK*) gene. This mutation does not, as its primary effect, eliminate a protein product required for maintenance of skeletal muscle. Instead, the trinucleotide repeat gives rise to deleterious RNAs that carry long tracts of CUG repeats. The propensity of this repetitive RNA to self-associate, together with its high affinity for splicing factors in the muscleblind-like (MBNL) family, drives the formation of RNA-protein condensates, called foci, in the nucleus (5, 6). When MBNL proteins become sequestered in the foci, many changes of the transcriptome ensue (7). For example, certain transcripts revert to fetal or nonmuscle patterns of alternative splicing, which compromises muscle function through misexpression of protein isoforms. Similar problems occur in other tissues expressing the expanded CUG repeats: smooth muscle, brain, and the heart (8, 9).

**Figure 1:**
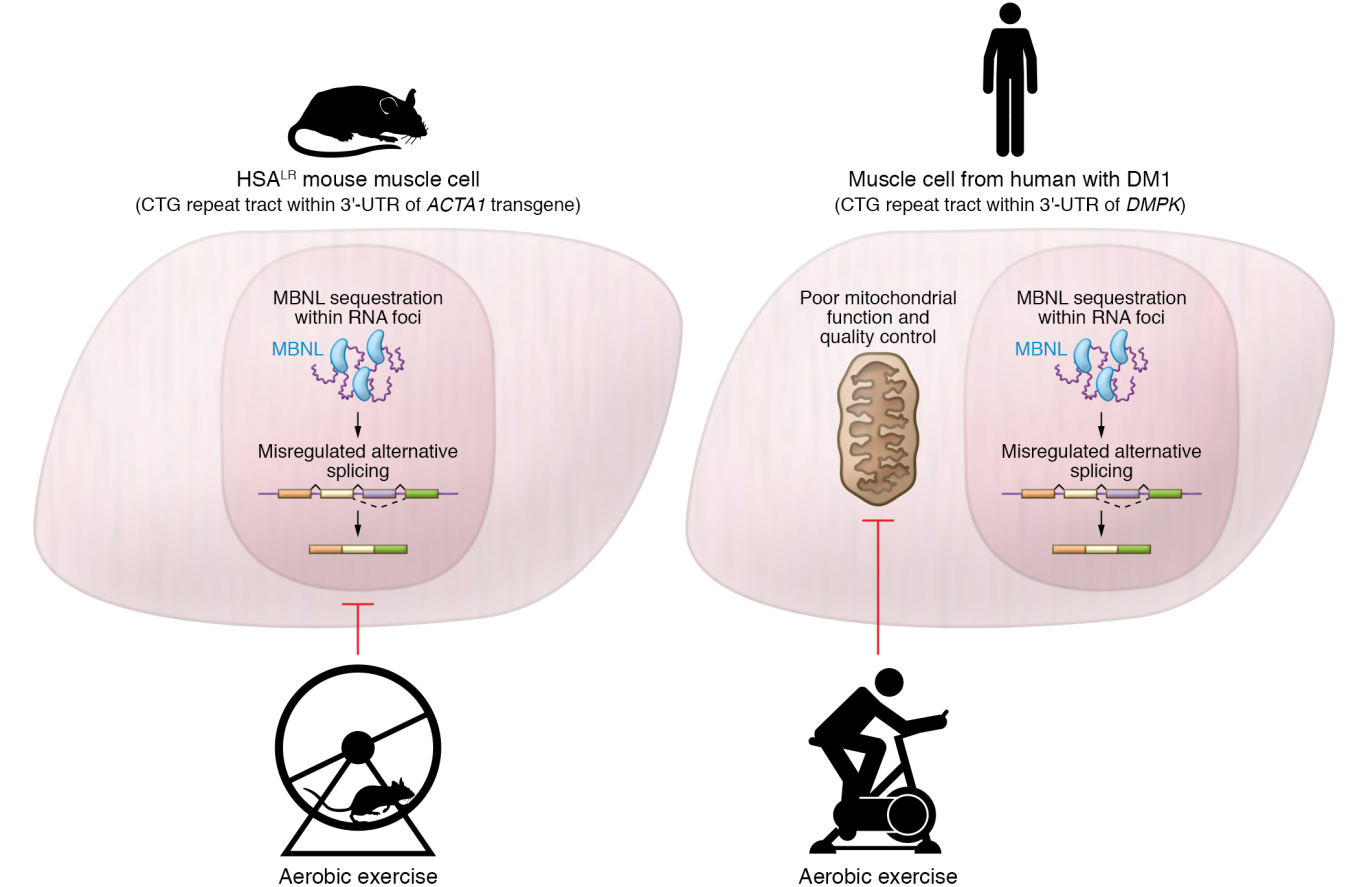
Aerobic exercise produces functional gains in HSA^LR^ transgenic mice and humans with DM1. While mice exhibit reductions of RNA foci and improvements in the regulation of alternative splicing, similar effects were not seen in humans with DM1. Increased mitochondrial function and quality control may explain the benefits of exercise in DM1-affected individuals. *ACTA1*, actin α-1.

Although current theories about RNA toxicity offer no indication as to whether DM1 muscles may have increased sensitivity to activity-induced damage or heightened responsivity to exercise, four separate studies indicate that exercise is safe and highly beneficial in a transgenic DM1 mouse model, acting to reduce RNA toxicity and partially restore the transcriptome (10–13). Reporting in this issue of the *JCI*, Mikhail and colleagues test the translation of this finding in patients with DM1, and show that exercise is indeed beneficial, but not in ways predicted by studies in transgenic mice (Figure 1 and ref. 14). The authors examined the effects of supervised aerobic exercise on a cycle ergometer in 11 participants with DM1. Although the intensity of the training was considered moderate (30 minutes, 3 times weekly for 12 weeks at 65%–75% maximal capacity), the subjects showed impressive gains. For example, maximum oxygen consumption, which at baseline was only 52% that of healthy controls, improved by 32%. Functional mobility measures, such as six-minute walk distance, showed parallel improvements, and lean body mass increased 1.6 kg on average. However, in contrast to what occurred in transgenic mice, the physiological improvements in DM1 patients were not accompanied by decreased RNA toxicity, as determined by analysis of RNA foci, MBNL proteins, and splicing regulation in pre- and postexercise biopsy samples of quadriceps muscle.

## Difference of molecular response in mice and humans

What accounts for the disparity between mice and humans? One possibility lies in the intensity of the exercise stimulus, which was more frequent and of longer duration in mice than in patients. Another reason may relate to differences in the size of expanded repeats. The CTG repeat in the transgenic mouse model (designated HSA^LR^) is unstable, often hovering around 220 repeats, which is near the theoretical threshold for robust nuclear retention and formation of foci. If exercise promotes the nuclear export of CUG-repeat transcripts that are loosely held in foci, this effect may be more pronounced in mice than in patients, who typically carry several thousand CTG repeats in muscle tissue and whose nuclear foci appear far more condensed than those observed in HSA^LR^ mice.

The simplest explanation, however, may relate to the impact of exercise on the accumulation of toxic RNA from the gene harboring the repeats. In the transgenic mouse model, a genomic fragment containing the entire human skeletal actin gene, with an expanded CTG repeat in the 3′ UTR, was integrated in the mouse genome to drive high expression of CUG repeats in skeletal muscle (15). Sharp and colleagues showed that treadmill exercise can reduce the level of mRNA from this transgene. Since expression of the endogenous mouse skeletal actin gene was not affected, they postulated that exercise increased the degradation of transgene mRNA (10).

In fact, these explanations may coincide. In a previous study of the same mouse model, an oligonucleotide drug was used to disperse foci and increase the nuclear export of CUG-repeat RNA. Unexpectedly, this intervention also caused substantial downregulation of the transgene mRNA via accelerated turnover (16). It is noteworthy, however, that Mikhail and colleagues observed no downregulation of *DMPK* mRNA following exercise, which may reflect differences of *DMPK* regulation or the difficulty of forcing nuclear export when the repeat tract is extremely long (14).

## Mitochondrial function and snoRNAs in DM1

If the benefits of exercise in DM1 patients do not involve reduction of RNA toxicity, then what is the mechanism? Mikhail et al. present evidence that RNA toxicity, though not improved by exercise, does not block normal adaptations of muscle to aerobic training (14). In line with previous work (17, 18), Mikhail and colleagues found that mitochondrial function was impaired at baseline in DM1 muscle. The authors then went on to demonstrate several of the expected improvements from endurance training. For example, proteins in complexes I, III, and IV of the respiratory chain were reduced at baseline and restored after exercise.

RNA-Seq of DM1 muscle also showed a huge upregulation of small nucleolar RNAs (snoRNAs), which are noncoding RNAs involved in ribosome biogenesis and other aspects of RNA metabolism (19, 20). This programmatic shift of an entire class of small RNAs was unexpected, and the functional importance is unclear. If confirmed, snoRNAs may provide useful biomarkers of the disease process. However, further work is needed to determine how this alteration is linked to the RNA gain-of-function mechanism and whether it is a direct consequence contributing to molecular pathology or part of a compensatory response.

## Clinical implications and future directions

Although large well-controlled studies are lacking, the report by Mikhail et al. (14) adds to the existing evidence that limb muscles in DM1 can respond favorably to resistance and endurance exercise (21). Certainly, it is premature to conclude that exercise in DM1 is uniformly safe, especially considering the heterogeneity of the disease. However, the authors tested for signs of aggravated muscle damage, assessing muscle histology and serum creatine kinase, and found none (14). An unresolved question is whether the pre-exercise reduction of mitochondrial function and aerobic capacity represents a primary effect of the disease process or whether these changes result mainly from severe deconditioning in a disease in which fatigue, limited mobility, daytime hypersomnolence, muscle pain, and other factors often conspire to produce a sedentary lifestyle. The finding that moderate aerobic exercise in humans did not fundamentally modify either the level of toxic RNA or extent of MBNL sequestration may not necessarily apply to resistance training, and it remains possible that the short-term benefits of aerobic exercise may still translate to long-term myoprotective effects, if adherence to a regimen can be sustained (14). Going forward, it will be important to find the optimal regimens of aerobic and resistance exercise that safely deliver benefits and develop strategies to maximize adherence in this population (22).

## Version 1 05/16/2022

Electronic publication
